# Neoplastic Progression Risk in Females With Barrett’s Esophagus: A Systematic Review and Meta-Analysis of Individual Patient Data

**DOI:** 10.1016/j.cgh.2024.06.053

**Published:** 2024-10-05

**Authors:** Pauline A. Zellenrath, Laurelle van Tilburg, Roos E. Pouw, Rena Yadlapati, Yonne Peters, Michael B. Ujiki, Prashanthi N. Thota, Norihisa Ishimura, Stephen J. Meltzer, Noam Peleg, Won-Tak Choi, John V. Reynolds, Alexandros D. Polydorides, Arjun D. Koch, Judith Honing, Manon C. W. Spaander

**Affiliations:** 1 Division of Gastroenterology and Hepatology, Erasmus MC Cancer Institute, Erasmus University Medical Center, Rotterdam, the Netherlands; 2 Department of Gastroenterology and Hepatology, Amsterdam University Medical Center, Amsterdam, the Netherlands; 3 Department of Gastroenterology and Hepatology, UC San Diego School of Medicine, San Diego, California; 4 Department of Gastroenterology and Hepatology, Radboud University Medical Center, Nijmegen, the Netherlands; 5 Department of Surgery, NorthShore University HealthSystem, Evanston, Illinois; 6 Department of Gastroenterology and Hepatology, Cleveland Clinic Foundation, Cleveland, Ohio; 7 Second Department of Internal Medicine, Shimane University Faculty of Medicine, Shimane, Japan; 8 Department of Gastroenterology and Hepatology, Johns Hopkins University School of Medicine, Baltimore, Maryland; 9 Devision of Gastroenterology and Hepatology, Rabin Medical Center, Petah-Tikva, Israel; 10 Department of Pathology, University of California, San Francisco, San Francisco, California; 11 Department of Surgery, Trinity Center for Health Sciences, St. James’s Hospital, Trinity College Dublin, Dublin, Ireland; 12 Department of Pathology, Molecular and Cell Based Medicine, Icahn School of Medicine at Mount Sinai, New York, New York

**Keywords:** Individual Patient Data, BE, Sex, Neoplastic Progression

## Abstract

**BACKGROUND AND AIMS:**

Females with Barrett’s esophagus (BE) have a lower risk of neoplastic progression than males, but sufficiently powered risk analyses are lacking. This systematic review and meta-analysis of individual patient data (IPD) aimed to provide more robust evidence on neoplastic progression risk in females.

**METHODS:**

We conducted a systematic literature search of 3 electronic databases (Medline, Embase, Google Scholar) from inception until August 2023. Eligible studies (1) reported original data on progression from nondysplastic BE, indefinite for dysplasia, or low-grade dysplasia to high-grade dysplasia or esophageal adenocarcinoma; and (2) included female and male patients. IPD were quality controlled by 2 independent reviewers. The primary outcome was the association between sex and neoplastic progression risk, adjusted for risk factors using multivariable Cox regression analysis. Secondary outcomes were sex differences in time to progression and annual progression rate.

**RESULTS:**

IPD were obtained from 11 of 66 eligible studies, including 2196 (31%) females. Neoplastic progression risk was lower in females (hazard ratio for males vs females, 1.44; 95% confidence interval, 1.13-1.82) after adjusting for age, smoking, medication use, hiatal hernia, BE length, and baseline pathology. The annual progression rate was 0.88% in females vs 1.29% in males. Time to progression was similar in both sexes: 3.7 years (interquartile range, 2.1-7.7 years) in females and 4.2 years (interquartile range, 2.0-8.1 years) in males.

**CONCLUSION:**

Although females had a lower neoplastic progression risk, sex differences were smaller than previously reported, and time to progression was similar for both sexes. Future research should focus on other factors than sex to identify low- and high-risk BE patients.

## Graphical Abstract

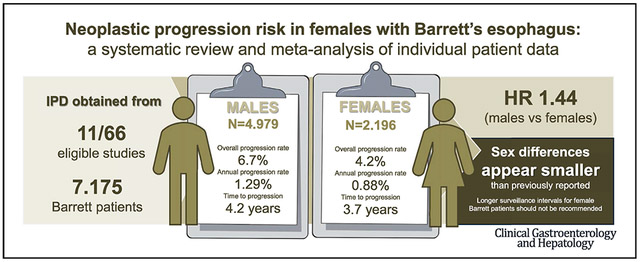

Barrett’s esophagus (BE) is a relatively common condition that predisposes to the development of esophageal adenocarcinoma (EAC), a malignancy with an overall 5-year survival rate of 21%.^1^ Frequent endoscopic surveillance of Barrett mucosa has been associated with detection of EAC in an early stage,^2^ allowing endoscopic removal with good outcomes. Therefore, endoscopic surveillance is currently recommended for all BE patients,^3–5^ while ideally surveillance should be limited to patients at high risk of progression in order to be cost-effective. ^6–7^

Several studies show that females with BE are less likely to develop neoplastic progression compared with males,^8–10^ which indicates that longer surveillance intervals for female patients might be justified. Nevertheless, current available data include low absolute numbers of female progressors. Thus, before safely excluding women from BE surveillance, a sufficiently powered multivariable risk analysis for neoplastic progression would be required. Melquist et al^11^ recently performed a systematic review and meta-analysis but did not provide true annual neoplastic progression rates for females from aggregated data and did not adjust for additional risk factors. With this systematic review with meta-analyses of individual patient data (IPD),^12^ we aimed to provide more robust evidence on neoplastic progression risk in females with BE compared with males, adjusted for known clinical and endoscopic risk factors.

## Materials and Methods

### Protocol and Registration

This systematic review and meta-analysis followed the PRISMA-IPD (Preferred Reporting Items for Systematic Reviews and Meta-analyses of IPD) reporting guidelines.^13^ The paper was written according to the Sex and Gender Equity in Research guidelines.^14^ The study protocol is registered on the PROSPERO (CRD42023450218).

### Eligibility criteria

Studies were eligible for inclusion if they (1) reported original data on progression rates from nondysplastic Barrett’s esophagus (NDBE), indefinite for dysplasia (IND), or low-grade dysplasia (LGD) to high grade dysplasia (HGD) or EAC; and (2) included both female and male patients. Exclusion criteria included (1) case reports/series and case-control studies, (2) studies restricted to specific BE diagnostics or treatments, (3) studies restricted to patients with a family history of BE/EAC, (4) articles not written in English, and (5) articles not published in full text. If patients were assumed to be duplicate in multiple articles, only the most recently published article was considered eligible for inclusion.

### Information Source and Search Strategy

We conducted a systematic search of electronic medical databases (Medline, Embase, and Google Scholar) from inception until the August 10, 2023. The search strategy was designed in close collaboration with an experienced biomedical information specialist and was specifically tailored for each database. The search query consisted of synonyms and thesaurus terms of 2 concepts: (1) BE and (2) neoplastic progression. The search was restricted to human studies with adult patients, and studies including at least 100 patients. There were no restrictions imposed on publication date of the study. Study registries were not searched, and we did not browse unindexed journals in the field. The full search strategy is available in Supplement 1.

### Study Selection

Records from the search were imported into an EndNote library through electronic extraction. After deduplication using the Bramer method,^15^ 2 reviewers (P.A.Z. and L.v.T.) manually identified and removed any remaining duplicates. Subsequently, the reviewers independently screened titles and abstracts to select potentially eligible studies. Full-text reports of potentially eligible studies were retrieved and assessed for eligibility. Any disagreement was addressed and resolved through discussion. References from eligible studies were scanned to identify additional relevant publications that were missed by the initial search.

### Data Collection Processes

IPD from eligible studies were requested if contact details from the principal investigator or corresponding author were available. All data were de-identified at source. Only studies from which we received the IPD were included for analysis. Aggregated data were not extracted from studies that could not share the IPD, as our primary study aim could not be answered with the use of aggregated data.

### Data Items

IPD regarding relevant clinical and endoscopic risk factors at baseline as well as surveillance data were requested from all eligible studies (Supplement 2). Follow-up time was measured as time from index endoscopy to the last endoscopy in nonprogressors, or as the time from index endoscopy to the first endoscopy where HGD or EAC was diagnosed in progressors. If needed, we converted original variables within IPD datasets into standardized variables, to ensure common scales and measurements across studies.

### IPD Integrity

Two authors (P.A.Z. and L.v.T.) independently assessed the original IPD datasets. The data were checked with respect to range, missing or extreme values, errors, and consistency with the published data. Details such as study design and inclusion criteria were cross-checked against the original publications. Inconsistencies or missing data were discussed and resolved with the collaborators.

### Quality Assessment and Risk of Bias

The Newcastle-Ottawa Scale checklist was used for the quality assessment of included studies. Two reviewers (P.A.Z. and L.v.T.) filled out the Newcastle-Ottawa Scale checklist for each study, with total scores of 0–3, 4–6, and 7–8 documented as low, moderate, or high quality, respectively. Any differences were resolved through discussion. Studies judged as low quality on all domains relating to bias or applicability were considered at high risk of bias. Also, if a minimum of 10 studies could be included, a funnel plot was made to evaluate for publication bias. A responder analysis was performed to assess the risk of bias relating to the accumulated body of evidence, including any pertaining to not obtaining IPD for particular studies.

### Outcomes

The primary outcome was the association between sex and neoplastic progression, measured as adjusted hazard ratio (HR), after adjusting for baseline variables associated with neoplastic progression in BE, such as age, body mass index (BMI), medication use, and endoscopic features, including the presence of an hiatal hernia, BE segment length, and pathology. Secondary endpoints were time to progression (reported as median time in years from index endoscopy to the endoscopy in which HGD or EAC was diagnosed for the first time), and annual progression rate.

### Data Synthesis and Statistical Analysis

IPD from individual studies were quality controlled and a study identification variable was added for each patient Subsequently, the IPD were merged for analysis. Patients were excluded from analysis if sex was unknown or if follow-up data were not available. Patients with baseline HGD/EAC or with an interval of <6 months between index endoscopy and neoplastic progression were excluded from analysis to prevent the inclusion of prevalent cases. Missing data were assumed to be missing at random. Multiple imputation was performed to adjust for missing values, using the Substantive Model Compatible Fully Conditional Specification multiple imputation method.^16^ Analyses were performed on the imputed datasets and then pooled using Rubin’s rules.^17^

Descriptive analyses on baseline demographic and clinical characteristics were conducted to characterize the patients included in our IPD analysis. Dichotomous variables were reported as percentage and continuous variables were reported as mean ± SD when normally distributed or as median (interquartile range [IQR]) when they were not normally distributed. Heterogeneity between included studies was assessed using the inconsistency index (*I*^2^). Excessive influence of individual studies on the pooled HR was investigated with a leave-one-out analysis. We used the one-step approach for the IPD meta-analysis and accounted for clustering of patients within studies by using a mixed effects Cox regression model that also included a frailty factor for the individual studies. Independent variables for the multivariable Cox proportional hazards model were selected based on available literature about neoplastic progression risk in BE patients.^8^ Information on competing outcomes for the development of neoplastic progression, such as death or endoscopic treatment of patients with LGD, were not available in the IPD dataset and could not be accounted for. The Kaplan-Meier method was used to visually summarize the association between sex and neoplastic progression risk over time. Publication bias was assessed using funnel plots, and asymmetry of the funnel was evaluated with an Egger regression test.

All analyses were performed using IBM SPSS statistics, version 25 (IBM), and R version 4.1.1 (R Foundation for Statistical Computing). Outcomes were reported with a 95% confidence interval (CI). Tests were considered statistically significant if *P* value was <.05 (2-sided).

### Role of the Funding Source

This study had no funding; therefore, there was no sponsor involved in any decisions regarding the study design, data collection, data analysis, data interpretation, or writing of the report. Three authors (M.C.W.S., P.A.Z., and L.v.T.) had full access to all the data in the study, and all authors had final responsibility for the decision to submit for publication.

## Results

### Study Selection and IPD obtained

**Figure 1.**
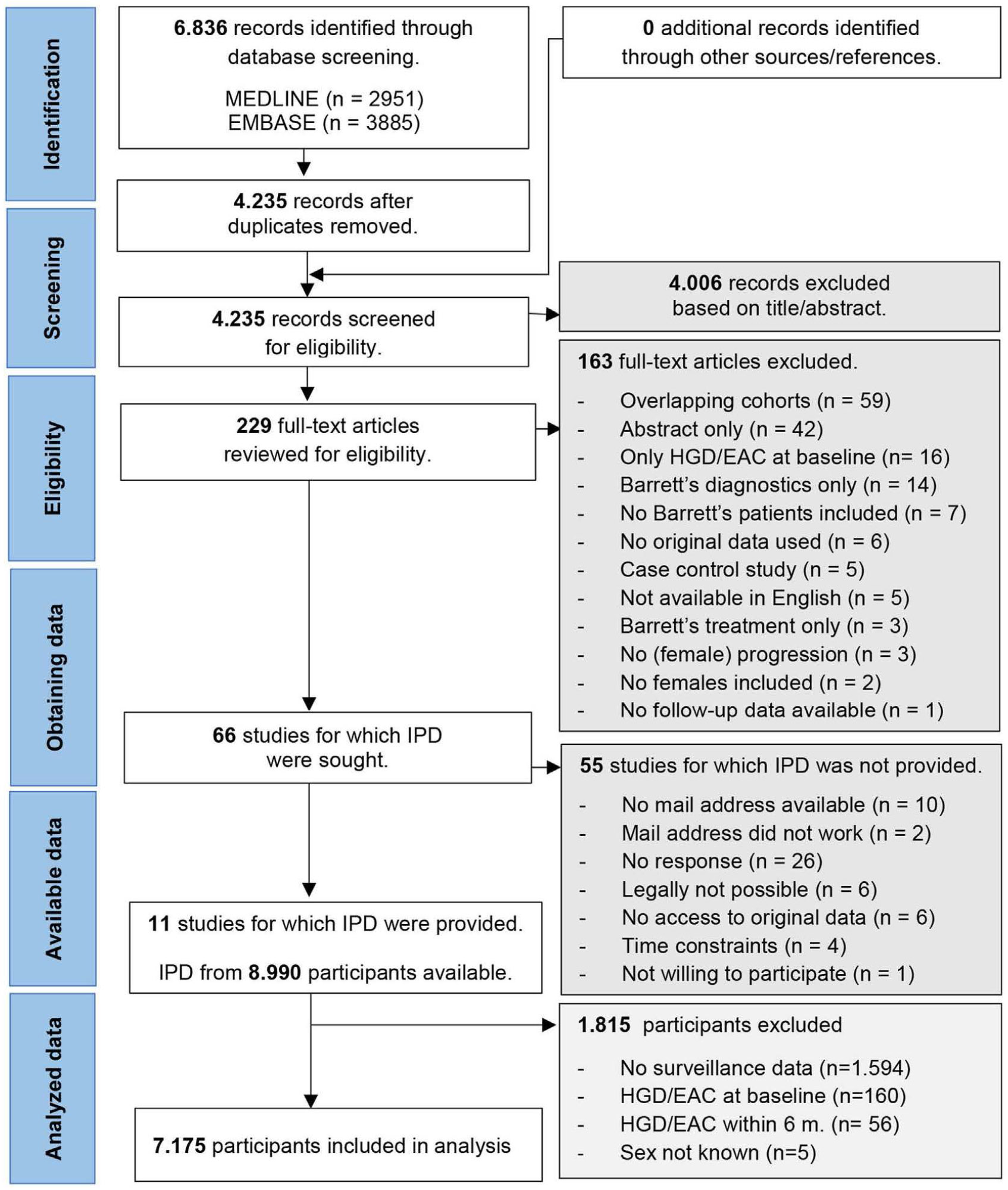
PRISMA-IPD flow diagram: the identification of studies via databases and registers.

A flow diagram of our study selection process is shown in Figure 1. In total, 66 articles met our inclusion criteria. IPD were successfully retrieved of 11 (17%) of 66 articles, including 8990 BE patients. A responder analysis is included in Supplement 3. We excluded 1815 patients from the merged dataset because no surveillance data were available (n = 1594), baseline pathology was HGD/EAC (n = 160), HGD/EAC was diagnosed within 6 months after baseline (n = 56), or sex was unknown (n = 5). Overall, IPD from 7175 individual patients were included for the meta-analyses, including 2196 (30.6%) females. Baseline characteristics of the included patients are shown in Table 1.

### Study Characteristics

Details of the 11 included studies are shown in Table 2.^9,18–27^ Overall, 7175 BE patients with baseline NDBE (86%), IND (5%), or LGD (9%) were included between October 1979 and June 2021. Median follow-up time in the included studies ranged from 1.9 to 7.9 years. The neoplastic progression rate in the included studies in females ranged from 0% to 19.9%, and the progression rate in males ranged from 0.4% to 34.4%.

### IPD Integrity and Risk of Bias Within Studies

Discrepancies between the provided IPD and the original reports were found in 2 of the 11 studies. All discrepancies were successfully resolved by correspondence with the authors. Included studies were rated as moderate-to-high quality, with scores ranging from 5 to 9 out of a maximum of 9 using the Newcastle-Ottawa Scale (Supplementary table 1). Therefore, all studies met the quality threshold for inclusion in the quantitative synthesis.

### Results of Individual Studies

**Figure 2.**
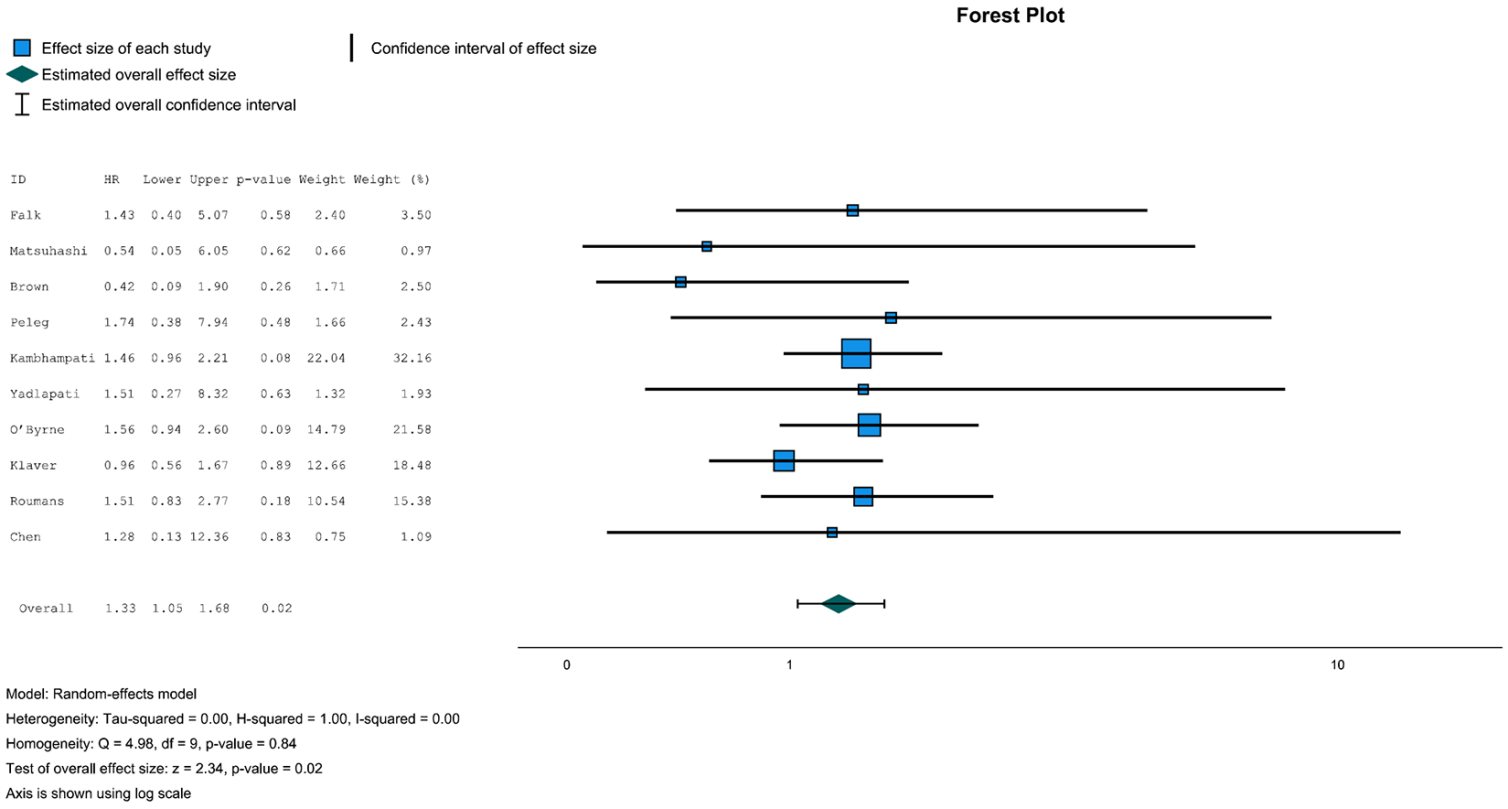
Pooled HR for neoplastic progression in females vs males with BE.

The pooled HR of males vs females for developing neoplastic progression was 1.33 (95% CI, 1.05–1.68; *P* = .02) (Figure 2). Heterogeneity among studies for this analysis was low (*I*^2^ = 0%). The cohort of Choi et al^21^ was excluded from the pooled analysis, as they did not report any female progressors. As the weight of Kambhampati et al^23^ was as high as 32% on this pooled analysis, we also performed a leave-one-out analysis. When excluding data from Kambhampati et al, the pooled HR of males vs females for developing neoplastic progression was 1.27 (95% CI, 0.96–1.69; *P* = .10) (Supplementary Figure 1). Heterogeneity among studies for this analysis was also low (*I*^2^ = 0%).

### Results of Data Syntheses (Primary Aim)

The median follow-up time within the complete IPD cohort was 3.8 years (IQR, 2.1–6.7 years). Overall, neoplastic progression was found in 92 (4.2%; 95% CI, 3.4%–5.1%) of 2196 females and in 333 (6.7%; 95% CI, 6.0%–7.4%) of 4979 males (Table 3). In females specifically, progression was found in 3.4% of patients with baseline NDBE, 3.5% with IND, and 13.2% with LGD. In males, progression was found in 5.5% of patients with baseline NDBE, 8.7% with IND, and 17.5% with LGD. Median time to progression was similar in both sexes: 3.7 years (IQR, 2.1–7.7 years) in the female cohort and 4.2 years (IQR, 2.0–8.1 years) in the male cohort The incidence rate of progression to HGD/EAC was 0.88% (95% CI, 0.71%–1.08%) per person-year in females and 1.29% (95% CI, 1.16%–1.44%) per person-year in males, with a combined overall incidence rate of 1.17% per person-year. The incidence rate ratio was 1.46 (95% CI, 1.16–1.86; *P* < .001) for males vs females. Kaplan Meier curves for progression-free survival were significantly different between males and females (chi-square = 8.831, *P* = .003) (Supplementary Figure 2).

In a multivariable Cox regression model, male sex was significantly associated with an increased risk of neoplastic progression compared with female sex (HR, 1.44; 95% CI, 1.13–1.82; *P* = .003) (Table 4). The other risk factors in the multivariable model that were significantly associated with an increased risk of progression were an older age at first endoscopy (HR, 1.03; 95% CI, 1.02–1.04; *P* < .001), a higher BMI (HR, 1.04; 95% CI, 1.01–1.06; *P* = .002), (a history of) smoking (HR, 1.56; 95% CI, 1.21–2.01; *P* = .001), a longer BE segment length in centimeters (HR, 1.13; 95% CI, 1.09–1.16; *P* < .001), and a diagnosis of IND (HR, 2.35; 95% CI, 1.47–3.78; *P* < .001) or LGD (HR, 3.62; 95% CI, 2.82–4.64; *P* < .001) at baseline. Proton pump inhibitor use, aspirin use, and the presence of an hiatal hernia were not significantly associated with the risk of neoplastic progression. Similar predictive factors were found when looking at females specifically (Table 4]. However, a higher BMI was not a significant risk factor for progression in females. Additionally, aspirin use was associated with a reduced risk of neoplastic progression in females (HR, 0.69, 95% 0.11–0.71; *P* = .009).

### Risk of Bias Across Studies

This study includes IPD from 17% of all eligible studies. The responder analysis is available in Supplement 3. Visual inspection of the funnel plot and results of the Egger’s test (*P* = .095) showed no clear indication of publication bias based on the included studies (Supplementary Figure 3, Supplementary Table 2).

## Discussion

This systematic review and IPD meta-analysis is the largest study to date to assess potential sex differences in neoplastic progression in BE patients. Including surveillance data from 2196 (31%) female and 4979 male patients, we found a significantly lower risk of neoplastic progression in females (HR for males vs females, 1.44). Moreover, the annual neoplastic progression rate was significantly lower in females compared with males (0.88% vs 1.29%), while median time to progression was similar in females (3.7 years [IQR, 2.1–7.7 years]) compared with males (4.2 years [IQR, 2.0–8.1 years]).

While the lower neoplastic progression risk in females we found in this study is in line with results from previous studies, sex differences in neoplastic progression risk may be smaller than previously reported. A meta-analysis by Krishnamoorthi et al,^8^ which studied factors associated with neoplastic progression in BE, reported a neoplastic progression risk more than twice as high in males compared with females (odds ratio [OR], 2.16; 95% CI, 1.84–2.53; *I*^2^ = 0%). A recently published systematic review by Melquist et al^11^ specifically studying sex differences in neoplastic progression in BE confirmed these findings with a pooled OR of 0.44 in females vs males (95% CI, 0.30–0.65; *I*^2^ = 22%), which translates to an OR of 2.27 for males vs females. In our IPD meta-analysis, however, the risk difference was considerably smaller (HR for males compared with females, 1.44; 95% CI, 1.13–1.82; *P* = .003). Moreover, when performing a sensitivity analysis by leaving out the results from Kambhampati et al,^23^ which reported remarkably high progression rates, we did not find a significant difference in the risk of progression between sexes.

Melquist et al^11^ reported a lower annual neoplastic progression rate of 0.62% (95% Cl, 0,22%–l,75%; I^2^ = 90%) in females compared with 0.88% in our data, and a higher annual progression rate of 1.54% (95% CI, 0.83%–2.81%; I^2^ = 96%) in males compared with 1.29% in our data. This discrepancy may be caused by the fact that Melquist et al could only calculate annual progression rates based on the overall follow-up from included studies, while we could specify follow-up time per sex. Therefore, we believe that our IPD-based results, in which annual progression rates of females and males were relatively closer to each other, are more representative of the true progression rates. Additionally, while a previous cohort study showed a longer time to progression in females,^9^ our large IPD dataset showed that the time to progression is similar in female and male progressors. These results are important findings that would hinder the safe implementation of longer surveillance intervals for females with BE.

In our multivariable risk analysis, predictive factors for neoplastic progression in females were almost identical to the predictive factors that we found in the complete (mainly male) IPD cohort. These included an older age at first endoscopy, [a history of) smoking, a longer BE segment length in centimeters, and a diagnosis of IND or LGD at baseline. BMI was not associated with risk of progression in females, while it was significantly associated with an increased risk of progression in the complete cohort. This may be caused by the distribution of body fat, as men are more likely to have central fat distribution, which is associated with an increased risk of progression to cancer based on its proinfiammatory qualities.^28^ However, none of the included studies reported a waist-to-hip ratio, so we could not further investigate this association. Additionally, aspirin use was associated with a reduced risk of neoplastic progression in females but not in the complete cohort. A previous study also reported on the chemopreventive effect of aspirin in females but did not compare their results with males.^29^ It may be interesting to further investigate the protective effect of aspirin in females, but it must be noted that we had a high percentage of missing data regarding aspirin use, and even within the available data it is notoriously difficult to collect reliable information about medication use. Additionally, assessing a causal association between medication use and the risk of neoplastic progression would require information on medication use over a longer period of time, while we could only report at baseline. Therefore, our results on medication use in relation to the risk of neoplastic progression must be interpreted with care.

In summary, while it has often been suggested to tailor BE surveillance intervals based on sex due to low progression rates in females, our results provide more nuance in the discussion whether surveillance intervals should be longer for all female patients with BE. Especially the similar time to progression in both sexes is concerning, as long as we are not able to identify those females at high risk of progression for which longer surveillance intervals would not be safe. We therefore recommend that future research focus on other risk factors than sex to improve the risk stratification in BE patients and make surveillance programs more cost-effective. It must be noted that provided IPD was largely heterogeneous regarding the absolute risk of neoplastic progression. As our study period includes the 1990s in which low-resolution endoscopes were used, subtle abnormalities or early neoplastic lesions in the BE segment could have been missed. This could lead to inclusion of patients with prevalent advanced neoplasia at baseline that was missed at initial endoscopy, possibly explaining the remarkably high progression rate among some included studies.^21,23^ On the contrary, we expect this to have influenced results from females and males equally and therefore should not have influenced the risk difference between sexes. It would be useful to repeat our analyses with a large prospective dataset to further assess the true difference in the risk of progression between females and males.

Our study has several strengths. The central collection and analysis of IPD gave us the ability to deal appropriately with missing data and time-to-event data, allowing us to perform robust multivariable Cox regression analyses in an exceptionally large cohort of BE patients. Most importantly, our study includes more than 2000 females with BE (including almost 100 pro-gressors), enabling us to assess individual risk factors for neoplastic progression in females for the first time. As IPD were provided from BE patients around the world, our results are generally applicable for a large BE population. However, it must be noted that due to this worldwide participation, included studies adhered to different societal BE surveillance guidelines. In order to separate individual patient-level interactions from any trial-level interactions, we accounted for the clustering of patients within studies.^30^

Some limitations of this study should also be acknowledged. Most importantly, it was not possible to obtain all eligible IPD, as a considerable number of authors could not be traced or no longer had access to the data. Individual patient data from 55 of 66 studies were not provided, which must be taken into account when considering the results of this study. Not including these studies could introduce bias, although the results are not expected to change significantly for our specific research question. We put considerable effort into maximizing participation from authors, but the reasons for nonresponse are unclear. Despite this drawback, our study still benefits from a large sample size, allowing robust statistical analyses, and we believe our findings provide meaningful insights into sex differences in neoplastic progression risk in BE patients. We would expect other investigators attempting IPD to encounter similar issues and not be able to reach significantly different rates than our 17% response rate. Another limitation was that not all confounding parameters were present within the included studies. Some potentially relevant factors, such as aberrant p53 expression, were not available from most studies, and could therefore not be included in our risk analysis. This issue is merely to overcome with prospective data.

## Conclusions

The results of this IPD meta-analysis show that females with BE have a lower risk of neoplastic progression compared with males. However, the difference in neoplastic progression risk between males and females appear to be smaller than previously reported, ranging from 1.44 in the current study to 2.27 in a recent metaanalysis. While overall progression rates are important, time to progression is more clinically relevant for surveillance recommendations. The time to progression appeared to be similar in female and male progressors, which may hinder the safe implementation of longer surveillance intervals for females. Whether BE surveillance intervals in females may be extended requires further investigation based on prospectively collected data. As sex may not be an appropriate factor to stratify BE patients into high- and low-risk groups, efforts to reduce the burden of current surveillance programs should mainly focus on other risk factors.

## Supplementary Material

1

Note: To access the supplementary material accompanying this article, visit the online version of *Clinical Gastroenterology and Hepatology* at www.cghjournal.org, and at http://doi.org/10.1016/j.cgh.2024.06.053.

## Figures and Tables

**Table 1.** Baseline Characteristics of Patients Included in the Main IPD Meta-Analyses

Variable	Females (n = 2196)	Males (n = 4979)	Total (N = 7175)
Age, y	62 ±13	59 ± 13	60 ±13
Missing	0(0)	0(0)	0(0)
BMI, kg/m^2^	28.0 ± 6.1	27.5 ± 4.6	27.7 ± 5.1
Missing	775 (35)	1660 (33)	2435 (32)
Current/former smoker	933 (51)	2680 (64)	3613 (60)
Missing	362 (17)	757(15)	679 (16)
PPI use	1185(96)	2549 (93)	3734 (94)
Missing	956 (44)	224 (45)	3200 (45)
Aspirin use	287 (23)	771 (27)	1058 (26)
Missing	962 (44)	2139 (43)	3101 (43)
Hiatal hernia	738 (64)	1725(67)	2464 (66)
Missing	1040(47)	2413 (49)	3453 (48)
BE length^a^, cm	2(1–5)	3(2–5)	3 (2–5)
Missing	444 (20)	772 (16)	1215(16)
Baseline pathology			
NDBE	1921 (88)	4253 (86)	6174(86)
IND	113(5)	252 (5)	365 (5)
LGD	159(7)	446(9)	605(9)
Unspecified (max. LGD)	3(0.1)	28 (0.6)	31 (0.4)
Missing	0(0)	0(0)	0(0)
FU time, y	3.7 (2.0–6.3)	3.9 (2.1–6.8)	3.8 (2.1–6.7)
Missing	0(0)	0(0)	0 (0)
FU endoscopies	2(2–4)	3(2–5)	3(2—4)
Missing	835 (38)	1836 (37)	2671 (35)

Values are mean ± SD, n (%), or median (interquartile range).

BE, Barrett’s esophagus; BMI, body mass index; FU, foliow-up; IND, indefinite for dysplasia; IPD, individual patient data; LGD, low-grade dysplasia; NDBE, nandysplastic Barrett’s esophagus; PPI, proton pump Inhibitor.

a BE length provided as the maximum segment length.

**Table 2.** Study Characteristics of Studies That Provided IPD

Study	Year	Country	Study Design	Centers	Baseline Pathology	Follow-Up Period	Follow-Up (y)	Patients	Females		Males	
									Total	HGD/EAC	Total	HGD/EAC
Falk^18^	2005	United States	Prospective	1	NDBE/LGD	1979–2002	3.2 (1.8–5.5)	340	89	3 (3.4)	251	14 (5.6)
Matsuhashi^19^	2017	Japan	Prospective	34	NDBE	2010–2015	1.9(1.1–2.7)	132	25	1 (4.0)	107	2 (2.0)
Brown^20^	2018	United States	Retrospective	6	NDBE/LGD	2000–2013	4.0 (2.2–6.4)	1322	505	4 (0.8)	817	3 (0.4)
Choi^21^	2018	United States	Retrospective	1	IND/LGD	1990–2015	2.7(1.0–5.0)	43	8	0 (0.0)	35	8 (23)
Peleg^22^	2020	Israël	Prospective	1	NDBE/IND/LGD	2013–2018	4.2 (2.7–5,2)	311	80	2 (2.5)	231	10 (4.3)
Kambhampati^23^	2020	United States	Retrospective	1	NDBE/LGD	1992–2013	6.4 (3.0–10.0)	452	141	28 (19.9)	311	107 (34.4)
Yadlapati^24^	2020	United States	Retrospective	4	NDBE/LGD	2014–2018	3.0(1.3–5.0)	100	48	2 (4.2)	52	4(7.7)
O’Byrne^25^	2020	Ireland	Prospective	6	NDBE/IND/LGD	2008–2017	2.7(1.3–4.0)	2202	692	21 (3.0)	1510	75 (5.0)
KIaver^26^	2021	Netherlands	Prospective	6	NDBE/LGD	2003–2017	7.9 (4.1–12.4)	985	258	17 (6.6)	727	50 (6.9)
Roumans^9^	2022	Netherlands	Prospective	18	NDBE/IND/LGD	2003–2020	5.9 (3.1–10.7)	1142	302	13 (4.3)	840	57 (6.8)
Chen^27^	2023	United States	Retrospective	1	IND	2000–2021	2.9(1.6–4.8)	146	48	1 (2.1)	98	3 (3.1)

Values are median (interquartile range), n, or n (%).

BE, Barrett’s eosphagus; EAC, esophageal adenocarcinoma; HGD, high-grade dysplasia; IND, indefinite for dysplasia; IPD, individual patient data; LGD, low-grade dysplasia; NDBE, nondysplastic Barrett’s esophagus.

**Table 3.** Absolute Number of Male and Female Progressors

Neoplastic Progression	Females (n = 2196)	Males (n = 4979)	Total (N = 7175)
HGD	51 (2.3; 1.7–3.0)	191 (3.8; 3.3–4.4)	242 (3.4; 3.0–3.8)
EAC	41 (1.9; 1.3–2.5)	142 (2.9; 2.4–3.4)	183 (2.6; 2.2–2.9)
Total	92 (4.2; 3.4–5.1)	333 (6.7; 6.0–7.4)	425 (5.9; 5.4–6.5)

Values are n (%; 95% confidence interval).

EAC, esophageal adenocarcinoma; HGD, high-grade dysplasia.

**Table 4.** Multivariable Cox Regression Model to Calculate Neoplastic Progression Risk

	Complete Cohort			Females Only		
	HR	95% Cl	P Value^a^	HR	95% Cl	P Value^a^
Sex			.003^b^			
Female	1	—		—	—	
Male	1.44	1.13–1.82		—	—	
Age	1.03	1.02–1.04	<.001^b^	1.05	1.03–1.08	<001^b^
BMI	1.04	1.01–1.06	.002^b^	1.02	0.94–1.10	.58
Smoking			.001^b^			.017^b^
No smoker	1	—		1	—	
Former/current smoker	1.56	1.21–2.01		1.75	1.11–2.77	
PPI use			.24			.65
No	1	—		1	—	
Yes	1.70	1.21–2.02		0.69	0.11–4.24	
Aspirin use			.16			.009^b^
No	1	—		1	—	
Yes	0.76	0.52–1.13		0.28	0.11–0.71	
Hiatal hernia			.78			.22
No	1	—		1	—	
Yes	0.95	0.64–1.41		0.7	0.43–1.22	
BE length	1.13	1.09–1.16	<.001^b^	1.18	1.09–1.26	<.001^b^
Baseline pathology			<.001^b^			<.001^b^
NDBE	1	—		1	—	
IND	2.35	1.47–3.78		1.50	0.47–4.76	
LGD	3.62	2.82–4.64		3.42	1.91–6.13	

BE, Barrett’s eosphagus; BMI, body mass index; Cl, confidence interval; HR, hazard ratio; IND, indefinite for dysplasia; LGD, low-grade dysplasia; NDBE, non-dysplastic Barrett’s esophagus; PPI, proton pump Inhibitor.

a *P*-value of the obtained hazard rate.

b Statistically significant result.

## What You Need to Know

### Background

Previous studies have shown that female Barrett’s esophagus patients have a lower neoplastic progression risk than males, but sufficiently powered risk analyses are required before surveillance can be adjusted according to sex.

### Findings

This study, including 7175 Barrett’s esophagus patients, shows that sex differences in risk of neoplastic progression were considerably smaller than previously reported, and time to progression was similar in both sexes.

### Implications for patient care

Longer surveillance intervals for female Barrett’s esophagus patients should not be recommended, and other risk factors than sex should be used to stratify Barrett’s esophagus patients for the risk of neoplastic progression.

## Abbreviations used in this paper:

BE: Barrett’s esophagus
BMI: body mass index
CI: confidence interval
EAC: esophageal adenocarcinoma
HGD: high-grade dysplasia
HR: hazard ratio
IND: indefinite for dysplasia
IPD: individual patient data
IQR: interquartile range
LGD: low-grade dysplasia
NDBE: nondysplastic Barrett’s esophagus
OR: odds ratio
