# Supplementary material for: Neoplastic Progression Risk in Females With Barrett’s Esophagus: A Systematic Review and Meta-Analysis of Individual Patient Data
Source: Clin Gastroenterol Hepatol. Author manuscript; Available in PMC 2026 May 30. (PMC13222059; doi:10.1016/j.cgh.2024.06.053)
Supplement: 1 [file NIHMS2174240-supplement-1.pdf]

Supplement 1. Search Strategy

20-04-2021: Barrett esophagus gender difference
Risk malignant progression high grade/ adenocarcinoma

('Barrett esophagus'/de OR (Barrett* NEAR/3 (esophag* OR oesophag*)):ab,ti) AND ('sex difference'/ exp OR 'sex factor'/de OR (((sex OR gender*) NEAR/3 (difference* OR factor*)) OR ((men OR man OR male) NEAR/3 (female OR women OR woman) NEAR/6 (difference* OR factor* OR risk*))):ab,ti OR (sex OR gender* OR ((men OR man OR male) NEAR/3 (female OR women OR woman))):ti)

Embase.com	3267	3230
Medline ALL Ovid	2676	484
Total	5943	3714

Embase.com 3267

('Barrett esophagus'/de OR (('columnar epithelium'/ de OR 'intestine metaplasia'/exp) AND ('esophagus disease'/exp OR 'esophagus'/exp)) OR (Barrett* OR ((Intestinal-metaplas* OR column*-lined OR column*-metaplasia) NEAR/3 (esophag* OR oesophag*))):ab,ti) AND ('cohort analysis'/de OR 'longitudinal study'/de OR 'prospective study'/de OR 'retrospective study'/de OR 'observational study'/de OR epidemiology/exp OR 'epidemiological data'/exp OR 'follow up'/de OR risk/ exp/mj OR 'risk factors'/exp/mj OR (cohort* OR longitudinal* OR prospectiv* OR retrospectiv* OR surveillance OR (observation* NEAR/3 stud*) OR epidemiolog* OR prevalence* OR incidence* OR (risk NEAR/3 (malignan* OR tumor* OR tumour* OR cancer* OR neoplas* OR dysplas* OR adenocarcinom* OR progression* OR high-grade)) OR follow-up OR predict*):ab,ti) AND ('malignant neoplasm'/exp OR 'esophagus tumor'/exp OR 'high grade dysplasia'/de OR adenocarcinoma/de OR 'cancer risk'/de OR 'disease exacerbation'/de OR (malignan* OR tumor* OR tumour* OR cancer* OR neoplas* OR dysplas* OR adenocarcinom* OR progression* OR high-grade OR exacerbat*):ab,ti) AND (((50 OR 51 OR 52 OR 53 OR 54 OR 55 OR 56 OR 57 OR 58 OR 59 OR 60 OR 61 OR 62 OR 63 OR 64 OR 65 OR 66 OR 67 OR 68 OR 69 OR 70 OR 71 OR 72 OR 73 OR 74 OR 75 OR 76 OR 77 OR 78 OR 79 OR 80 OR 81 OR 82 OR 83 OR 84 OR 85 OR 86 OR 87 OR 88 OR 89 OR 90 OR 91 OR 92 OR 93 OR 94 OR 95 OR 96 OR 97 OR 98 OR 99 OR 00? OR 01? OR 02? OR 03? OR 04? OR 05? OR 06? OR 07? OR 08? OR 09? OR 10? OR 11? OR 12? OR 13? OR 14? OR 15? OR 16? OR 17? OR 18? OR 19? OR 20? OR 21? OR 22? OR 23? OR 24? OR 25? OR 26? OR 27? OR 28? OR 29? OR 30? OR 31? OR 32? OR 33? OR 34? OR 35? OR 36? OR 37? OR 38? OR 39? OR 40? OR 41? OR 42? OR 43? OR 44? OR 45? OR 46? OR 47? OR 48? OR 49? OR 50? OR 51? OR 52? OR 53? OR 54? OR 55? OR 56? OR 57? OR 58? OR 59? OR 60? OR 61? OR 62? OR 63? OR 64? OR 65? OR 66? OR 67? OR 68? OR 69? OR 70? OR 71? OR 72? OR 73? OR 74? OR 75? OR 76? OR 77? OR 78? OR 79? OR 80? OR 81? OR 82? OR 83? OR 84? OR 85? OR 86? OR 87? OR 88? OR 89? OR 90? OR 91? OR 92? OR 93? OR 94? OR 95? OR 96? OR 97? OR 98? OR 99? OR 10?? OR 11?? OR 12?? OR 13?? OR 14?? OR 15?? OR 16?? OR 17?? OR 18?? OR 19?? OR 20?? OR 21?? OR 22?? OR 23?? OR 24?? OR 25?? OR 26?? OR 27?? OR 28?? OR 29?? OR 30?? OR 31?? OR 32?? OR 33?? OR 34?? OR 35?? OR 36?? OR 37?? OR 38?? OR 39?? OR 40?? OR 41?? OR 42?? OR 43?? OR 44?? OR 45?? OR 46?? OR 47?? OR 48?? OR 49?? OR 50?? OR 51?? OR 52?? OR 53?? OR 54?? OR 55?? OR 56?? OR 57?? OR 58?? OR 59?? OR 60?? OR 61?? OR 62?? OR 63?? OR 64?? OR 65?? OR 66?? OR 67?? OR 68?? OR 69?? OR 70?? OR 71?? OR 72?? OR 73?? OR 74?? OR 75?? OR 76?? OR 77?? OR 78?? OR 79?? OR 80?? OR 81?? OR 82?? OR 83?? OR 84?? OR 85?? OR 86?? OR 87?? OR 88?? OR 89?? OR 90?? OR 91?? OR 92?? OR 93?? OR 94?? OR 95?? OR 96?? OR 97?? OR 98?? OR 99??) NEXT/6 (patients OR patient OR subjects OR individuals OR cases OR persons OR men OR women OR males OR females OR participant* OR people OR children OR adolescent* OR boys OR girls OR teens OR teenagers OR infants OR newborns OR elderly OR survivor* OR specimen* OR sample* OR episode* OR isolate* OR pediatric OR paediatric OR adult* OR esophag* OR oesophag*)) OR ((fifty* OR sixty* OR seventy* OR eighty* OR ninety* OR hundred OR thousand OR million) NEXT/5 (patients OR patient OR subjects OR individuals OR cases OR persons OR men OR women OR participants OR people OR children OR adolescent* OR boys OR girls OR teens OR teenagers OR infants OR newborns OR elderly OR survivor* OR specimen* OR sample* OR episode* OR isolate* OR pediatric OR paediatric OR esophag* OR oesophag*)) OR ((n OR included OR recruited OR randomized OR randomized OR assigned OR a-total-of) NEXT/2 (50 OR 51 OR 52 OR 53 OR 54 OR 55 OR 56 OR 57 OR 58 OR 59 OR 60 OR 61 OR 62 OR 63 OR 64 OR 65 OR 66 OR 67 OR 68 OR 69 OR 70 OR 71 OR 72 OR 73 OR 74 OR 75 OR 76 OR 77 OR 78 OR 79 OR 80 OR 81 OR 82 OR 83 OR 84 OR 85 OR 86 OR 87 OR 88 OR 89 OR 90 OR 91 OR 92 OR 93 OR 94 OR 95 OR 96 OR 97 OR 98 OR 99 OR 00? OR 01? OR 02? OR 03? OR 04? OR 05? OR 06? OR 07? OR 08? OR 09? OR 10? OR 11? OR 12? OR 13? OR 14? OR 15? OR 16? OR 17? OR 18? OR 19? OR 20? OR 21? OR 22? OR 23? OR 24? OR 25? OR 26? OR 27? OR 28? OR 29? OR 30? OR 31? OR 32? OR 33? OR 34? OR 35? OR 36? OR 37? OR 38? OR 39? OR 40? OR 41? OR 42? OR 43? OR 44? OR 45? OR 46? OR 47? OR 48? OR 49? OR 50? OR 51? OR 52? OR 53? OR 54? OR 55? OR 56? OR 57? OR 58? OR 59? OR 60? OR 61? OR 62? OR 63? OR 64? OR 65? OR 66? OR 67? OR 68? OR 69? OR 70? OR 71? OR 72? OR 73? OR 74? OR 75? OR 76? OR 77? OR 78? OR 79? OR 80? OR 81? OR 82? OR 83? OR 84? OR 85? OR 86? OR 87? OR 88? OR 89? OR 90? OR 91? OR 92? OR 93?

OR 94? OR 95? OR 96? OR 97? OR 98? OR 99? OR 10?? OR 11?? OR 12?? OR 13?? OR 14?? OR 15?? OR 16?? OR 17?? OR 18?? OR 19?? OR 20?? OR 21?? OR 22?? OR 23?? OR 24?? OR 25?? OR 26?? OR 27?? OR 28?? OR 29?? OR 30?? OR 31?? OR 32?? OR 33?? OR 34?? OR 35?? OR 36?? OR 37?? OR 38?? OR 39?? OR 40?? OR 41?? OR 42?? OR 43?? OR 44?? OR 45?? OR 46?? OR 47?? OR 48?? OR 49?? OR 50?? OR 51?? OR 52?? OR 53?? OR 54?? OR 55?? OR 56?? OR 57?? OR 58?? OR 59?? OR 60?? OR 61?? OR 62?? OR 63?? OR 64?? OR 65?? OR 66?? OR 67?? OR 68?? OR 69?? OR 70?? OR 71?? OR 72?? OR 73?? OR 74?? OR 75?? OR 76?? OR 77?? OR 78?? OR 79?? OR 80?? OR 81?? OR 82?? OR 83?? OR 84?? OR 85?? OR 86?? OR 87?? OR 88?? OR 89?? OR 90?? OR 91?? OR 92?? OR 93?? OR 94?? OR 95?? OR 96?? OR 97?? OR 98?? OR 99??))):ab,ti NOT ([conference abstract]/lim AND [2000-2018]/py) NOT ([animals]/lim NOT [humans]/lim) NOT (juvenile/exp NOT adults/exp)

Medline ALL Ovid 2676

(Barrett Esophagus / OR (Barrett* OR ((Intestinal-metaplas* OR column*-lined OR column*-metaplasia) ADJ3 (esophag* OR oesophag*))).ab,ti.) AND (exp Cohort Studies / OR Observational Study/ OR exp Epidemiology / OR Epidemiological Monitoring / OR * risk/ OR * Risk Factors/ OR (cohort* OR longitudinal* OR prospectiv* OR retrospectiv* OR surveillance OR (observation* ADJ3 stud*) OR epidemiolog* OR prevalence* OR incidence* OR (risk ADJ3 (malignan* OR tumor* OR tumour* OR cancer* OR neoplas* OR dysplas* OR adenocarcinom* OR progression* OR high-grade)) OR follow-up OR predict*).ab,ti.) AND (Neoplasms / OR Esophageal Neoplasms / OR Adenocarcinoma / OR Disease Progression / OR (malignan* OR tumor* OR tumour* OR cancer* OR neoplas* OR dysplas* OR adenocarcinom* OR progression* OR high-grade OR exacerbat*).ab,ti.) AND (((5# OR 6# OR 7# OR 8# OR 9# OR 0## OR 1## OR 2## OR 3## OR 4## OR 5## OR 6## OR 7## OR 8## OR 9## OR 1### OR 2### OR 3### OR 4### OR 5### OR 6### OR 7### OR 8### OR 9###) ADJ6 (patients OR patient OR subjects OR individuals OR cases OR persons OR men OR women OR participant* OR people OR children OR adolescent* OR boys OR girls OR teens OR teenagers OR infants OR newborns OR elderly OR survivor* OR specimen* OR sample* OR episode* OR isolate* OR pediatric OR paediatric OR adult* OR esophag* OR oesophag*)) OR ((fifty* OR sixty* OR seventy* OR eighty* OR ninety* OR hundred* OR thousand*) ADJ6 (patients OR patient OR subjects OR individuals OR cases OR persons OR men OR women OR participant* OR people OR children OR adolescent* OR boys OR girls OR teens OR teenagers OR infants OR newborns OR elderly OR survivor* OR specimen* OR sample* OR episode* OR isolate* OR pediatric OR paediatric OR adult* OR esophag* OR oesophag*)) OR ((n OR included OR recruited OR randomized OR randomized OR assigned) ADJ2 (1 5# OR 6# OR 7# OR 8# OR 9# OR 0## OR 1## OR 2## OR 3## OR 4## OR 5## OR 6## OR 7## OR 8## OR 9## OR 1### OR 2### OR 3### OR 4### OR 5### OR 6### OR 7### OR 8### OR 9###)) OR large*-cohort*).ab,ti. NOT (exp animals/ NOT humans/) NOT ((exp child/ OR exp infant/ or adolescent/) NOT exp adult/)

10-08-2023 Barrett esophagus gender difference (update)

Risk malignant progression high grade/ adenocarcinoma

New references: 1059

Database searched	Platform	Years of coverage	Records	Records after duplicates removed
Medline ALL	Ovid	1946–Present	2951	2945
Embase	Embase.com	1971–Present	3885	1290
Total			6836	4235

No other database limits were used than those specified in the search strategies.

Embase.com 3885

('Barrett esophagus'/de OR (('columnar epithelium'/de OR 'intestine metaplasia'/exp) AND ('esophagus disease'/exp OR 'esophagus'/exp)) OR (Barrett* OR ((Intestinal-metaplas* OR column*-lined OR column*-metaplasia) NEAR/3 (esophag* OR oesophag*))):ab,ti,kw) AND ('cohort analysis'/de OR 'longitudinal study'/de OR 'prospective study'/de OR 'retrospective study'/de OR 'observational study'/de OR epidemiology/exp OR 'epidemiological data'/exp OR 'follow up'/de OR risk/exp/mj OR 'risk factors'/exp/mj OR (cohort* OR longitudinal* OR prospectiv* OR retrospectiv* OR surveillance OR (observation* NEAR/3 stud*) OR epidemiolog* OR prevalence* OR incidence* OR (risk NEAR/3 (malignan* OR tumor* OR tumour* OR cancer* OR neoplas* OR dysplas* OR adenocarcinom* OR progression* OR high-grade)) OR follow-up OR predict*):ab,ti,kw) AND ('malignant neoplasm'/exp OR 'esophagus tumor'/exp OR 'high grade dysplasia'/de OR adenocarcinoma/de OR 'cancer risk'/de OR 'disease exacerbation'/de OR (malignan* OR tumor* OR tumour* OR cancer* OR neoplas* OR dysplas* OR adenocarcinom* OR progression* OR high-grade OR exacerbat*):ab,ti,kw) AND (((50 OR 51 OR 52 OR 53 OR 54 OR 55 OR 56 OR 57 OR 58 OR 59 OR 60 OR 61 OR 62 OR 63 OR 64 OR 65 OR 66 OR 67 OR 68 OR 69 OR 70 OR 71 OR 72 OR 73 OR 74 OR 75 OR 76 OR 77 OR 78 OR 79 OR 80 OR 81 OR 82 OR 83 OR 84 OR 85 OR 86 OR 87 OR 88 OR 89 OR 90 OR 91 OR 92 OR 93 OR 94 OR 95 OR 96 OR 97 OR 98 OR 99 OR 00? OR 01? OR 02? OR 03? OR 04? OR 05? OR 06? OR 07? OR 08? OR 09? OR 10? OR 11? OR 12? OR 13? OR 14? OR

15? OR 16? OR 17? OR 18? OR 19? OR 20? OR 21? OR 22? OR 23? OR 24? OR 25? OR 26? OR 27? OR 28? OR 29? OR 30? OR 31? OR 32? OR 33? OR 34? OR 35? OR 36? OR 37? OR 38? OR 39? OR 40? OR 41? OR 42? OR 43? OR 44? OR 45? OR 46? OR 47? OR 48? OR 49? OR 50? OR 51? OR 52? OR 53? OR 54? OR 55? OR 56? OR 57? OR 58? OR 59? OR 60? OR 61? OR 62? OR 63? OR 64? OR 65? OR 66? OR 67? OR 68? OR 69? OR 70? OR 71? OR 72? OR 73? OR 74? OR 75? OR 76? OR 77? OR 78? OR 79? OR 80? OR 81? OR 82? OR 83? OR 84? OR 85? OR 86? OR 87? OR 88? OR 89? OR 90? OR 91? OR 92? OR 93? OR 94? OR 95? OR 96? OR 97? OR 98? OR 99? OR 10?? OR 11?? OR 12?? OR 13?? OR 14?? OR 15?? OR 16?? OR 17?? OR 18?? OR 19?? OR 20?? OR 21?? OR 22?? OR 23?? OR 24?? OR 25?? OR 26?? OR 27?? OR 28?? OR 29?? OR 30?? OR 31?? OR 32?? OR 33?? OR 34?? OR 35?? OR 36?? OR 37?? OR 38?? OR 39?? OR 40?? OR 41?? OR 42?? OR 43?? OR 44?? OR 45?? OR 46?? OR 47?? OR 48?? OR 49?? OR 50?? OR 51?? OR 52?? OR 53?? OR 54?? OR 55?? OR 56?? OR 57?? OR 58?? OR 59?? OR 60?? OR 61?? OR 62?? OR 63?? OR 64?? OR 65?? OR 66?? OR 67?? OR 68?? OR 69?? OR 70?? OR 71?? OR 72?? OR 73?? OR 74?? OR 75?? OR 76?? OR 77?? OR 78?? OR 79?? OR 80?? OR 81?? OR 82?? OR 83?? OR 84?? OR 85?? OR 86?? OR 87?? OR 88?? OR 89?? OR 90?? OR 91?? OR 92?? OR 93?? OR 94?? OR 95?? OR 96?? OR 97?? OR 98?? OR 99??) NEXT/6 (patients OR patient OR subjects OR individuals OR cases OR persons OR men OR women OR males OR females OR participant* OR people OR children OR adolescent* OR boys OR girls OR teens OR teenagers OR infants OR newborns OR elderly OR survivor* OR specimen* OR sample* OR episode* OR isolate* OR pediatric OR paediatric OR adult* OR esophag* OR oesophag*)) OR ((fifty* OR sixty* OR seventy* OR eighty* OR ninety* OR hundred OR thousand OR million) NEXT/5 (patients OR patient OR subjects OR individuals OR cases OR persons OR men OR women OR participants OR people OR children OR adolescent* OR boys OR girls OR teens OR teenagers OR infants OR newborns OR elderly OR survivor* OR specimen* OR sample* OR episode* OR isolate* OR pediatric OR paediatric OR esophag* OR oesophag*)) OR ((n OR included OR recruited OR randomized OR randomized OR assigned OR a-total-of) NEXT/2 (50 OR 51 OR 52 OR 53 OR 54 OR 55 OR 56 OR 57 OR 58 OR 59 OR 60 OR 61 OR 62 OR 63 OR 64 OR 65 OR 66 OR 67 OR 68 OR 69 OR 70 OR 71 OR 72 OR 73 OR 74 OR 75 OR 76 OR 77 OR 78 OR 79 OR 80 OR 81 OR 82 OR 83 OR 84 OR 85 OR 86 OR 87 OR 88 OR 89 OR 90 OR 91 OR 92 OR 93 OR 94 OR 95 OR 96 OR 97 OR 98 OR 99 OR 00? OR 01? OR 02? OR 03? OR 04? OR 05? OR 06? OR 07? OR 08? OR 09? OR 10? OR 11? OR 12? OR 13? OR 14? OR 15? OR 16? OR 17? OR 18? OR 19? OR 20? OR 21? OR 22? OR 23? OR 24? OR 25? OR 26? OR 27? OR 28? OR 29? OR 30? OR 31? OR 32? OR 33? OR 34? OR 35? OR 36? OR 37? OR 38? OR 39? OR 40? OR 41? OR 42? OR 43? OR 44? OR 45? OR 46? OR 47? OR 48? OR 49? OR 50? OR 51? OR 52? OR 53? OR 54? OR 55? OR 56? OR 57? OR 58? OR 59? OR 60? OR 61? OR 62? OR 63? OR 64? OR 65? OR 66? OR 67? OR 68? OR 69? OR 70? OR 71? OR 72? OR 73? OR 74? OR 75? OR 76? OR 77? OR 78? OR 79? OR 80? OR 81? OR 82? OR 83? OR 84? OR 85? OR 86? OR 87? OR 88? OR 89? OR 90? OR 91? OR 92? OR 93? OR 94? OR 95? OR 96? OR 97? OR 98? OR 99? OR 10?? OR 11?? OR 12?? OR 13?? OR 14?? OR 15?? OR 16?? OR 17?? OR 18?? OR 19?? OR 20?? OR 21?? OR 22?? OR 23?? OR 24?? OR 25?? OR 26?? OR 27?? OR 28?? OR 29?? OR 30?? OR 31?? OR 32?? OR 33?? OR 34?? OR 35?? OR 36?? OR 37?? OR 38?? OR 39?? OR 40?? OR 41?? OR 42?? OR 43?? OR 44?? OR 45?? OR 46?? OR 47?? OR 48?? OR 49?? OR 50?? OR 51?? OR 52?? OR 53?? OR 54?? OR 55?? OR 56?? OR 57?? OR 58?? OR 59?? OR 60?? OR 61?? OR 62?? OR 63?? OR 64?? OR 65?? OR 66?? OR 67?? OR 68?? OR 69?? OR 70?? OR 71?? OR 72?? OR 73?? OR 74?? OR 75?? OR 76?? OR 77?? OR 78?? OR 79?? OR 80?? OR 81?? OR 82?? OR 83?? OR 84?? OR 85?? OR 86?? OR 87?? OR 88?? OR 89?? OR 90?? OR 91?? OR 92?? OR 93?? OR 94?? OR 95?? OR 96?? OR 97?? OR 98?? OR 99??)):ab,ti,kw NOT ([conference abstract]/lim AND [2000-2020]/py) NOT ([animals]/lim NOT [humans]/lim) NOT (juvenile/exp NOT adults/exp)

Medline ALL Ovid 2951

(Barrett Esophagus / OR (Barrett* OR ((Intestinal-metaplas* OR column*-lined OR column*-metaplasia) ADJ3 (esophag* OR oesophag*))).ab,ti,kf.) AND (exp Cohort Studies / OR Observational Study/ OR exp Epidemiology / OR Epidemiological Monitoring / OR * risk/ OR * Risk Factors/ OR (cohort* OR longitudinal* OR prospectiv* OR retrospectiv* OR surveillance OR (observation* ADJ3 stud*) OR epidemiolog* OR prevalence* OR incidence* OR (risk ADJ3 (malignan* OR tumor* OR tumour* OR cancer* OR neoplas* OR dysplas* OR adenocarcinom* OR progression* OR high-grade)) OR follow-up OR predict*).ab,ti,kf.) AND (Neoplasms / OR Esophageal Neoplasms / OR Adenocarcinoma / OR Disease Progression / OR (malignan* OR tumor* OR tumour* OR cancer* OR neoplas* OR dysplas* OR adenocarcinom* OR progression* OR high-grade OR exacerbat*).ab,ti,kf.) AND (((5# OR 6# OR 7# OR 8# OR 9# OR 0## OR 1## OR 2## OR 3## OR 4## OR 5## OR 6## OR 7## OR 8## OR 9## OR 1### OR 2### OR 3### OR 4### OR 5### OR 6### OR 7### OR 8### OR 9###) ADJ6 (patients OR patient OR subjects OR individuals OR cases OR persons OR men OR women OR participant* OR people OR children OR adolescent* OR boys OR girls OR teens OR teenagers OR infants OR newborns OR elderly OR survivor* OR specimen* OR sample* OR episode* OR isolate* OR pediatric OR paediatric OR adult* OR esophag* OR oesophag*)) OR ((fifty* OR sixty* OR seventy* OR eighty* OR ninety* OR hundred* OR thousand*) ADJ6 (patients OR patient OR subjects OR individuals OR cases OR persons OR men OR women OR participant* OR people OR children OR adolescent* OR boys OR girls OR teens OR teenagers OR infants OR newborns OR elderly OR survivor* OR specimen* OR sample* OR episode* OR isolate* OR pediatric

OR paediatric OR adult* OR esophag* OR oesophag*)) OR ((n OR included OR recruited OR randomized OR randomized OR assigned) ADJ2 (1 5# OR 6# OR 7# OR 8# OR 9# OR 0## OR 1## OR 2## OR 3## OR 4## OR 5## OR 6## OR 7## OR 8## OR 9## OR 1### OR 2### OR 3### OR 4### OR 5### OR 6### OR 7### OR 8### OR 9###)) OR large*-cohort*).ab,ti,kf. NOT (exp animals/ NOT humans/) NOT ((exp child/ OR exp infant/ or adolescent/) NOT exp adult/) NOT ((congres* OR abstract*).pt. AND 1800:2020.(sa_year).)

Supplement 2. Collected Data Items

The following individual patient data were requested from all eligible studies: participant number, sex, date of birth, ethnicity, body mass index, smoking history, family history of Barrett's esophagus/esophageal adenocarcinoma, proton pump inhibitor use, aspirin use, date of first endoscopy, age at Barrett's esophagus diagnosis, histopathological diagnosis at baseline, length of Barrett's segment in centimeters, presence and size in centimeters of hiatal hernia, date of last endoscopy, last histopathological diagnosis, follow-up time in months, number of subsequent endoscopies, worst histopathological diagnosis during follow-up, vital status, and cause of death (if applicable). In case of progression to high-grade dysplasia/esophageal adenocarcinoma, additional individual patient data on the date of progression, tumor stage, treatment, and time of follow-up and number of subsequent endoscopies after progression were requested. The following additional data were collected from studies of which the individual patient data were available: year of publication, first author, country of origin, study design, and number of participating centers.

Supplement 3. Responder Analysis

Most studies were missing because we were not able to establish contact with the authors (58%). For example, we did not have valid contact details from 10 (15%) studies, as these were older studies (published between 1985 and 2004) and did not report an email address. Twenty-eight (42%) other authors did not respond to our emails. Furthermore, 6 authors had no access to their data. Six (9%) other authors were willing to share data but were not able to provide individual patient data due to legal constraints. One (1%) author was not willing to share data.

Supplementary References

1. Falk GW, Thota PN, Richter JE, et al. Barrett's esophagus in women: demographic features and progression to high-grade dysplasia and cancer. Clin Gastroenterol Hepatol 2005; 3:1089–1094.
2. Matsuhashi N, Sakai E, Ohata K, et al. Surveillance of patients with long-segment Barrett's esophagus: a multicenter prospective cohort study in Japan. J Gastroenterol Hepatol 2017; 32:409–414.
3. Brown CS, Lapin B, Goldstein JL, et al. Predicting progression in Barrett's esophagus: development and validation of the Barrett's Esophagus Assessment of Risk Score (BEAR Score). Ann Surg 2018;267:716–720.
4. Choi WT, Tsai JH, Rabinovitch PS, et al. Diagnosis and risk stratification of Barrett's dysplasia by flow cytometric DNA analysis of paraffin-embedded tissue. Gut 2018;67:1229–1238.
5. Peleg N, Schmilovitz-Weiss H, Shamah S, et al. Neutrophil to lymphocyte ratio and progression in patients with Barrett's esophagus. Endoscopy 2021;53:774–781.
6. Kambhampati S, Tieu AH, Luber B, et al. Risk factors for progression of Barrett's esophagus to high grade dysplasia and esophageal adenocarcinoma. Sci Rep 2020;10:4899.
7. Yadlapati R, Triggs J, Quader F, et al. Reduced esophageal contractility is associated with dysplasia progression in Barrett's esophagus: a multicenter cohort study. Dig Dis Sci 2020; 65:3631–3638.
8. O'Byrne LM, Witherspoon J, Verhage RJJ, et al. Barrett's Registry Collaboration of academic centers in Ireland reveals high progression rate of low-grade dysplasia and low risk from nondysplastic Barrett's esophagus: report of the RIBBON network. Dis Esophagus 2020;33:doaa099.
9. Klaver E, Bureo Gonzalez A, Mostafavi N, et al. Barrett's esophagus surveillance in a prospective Dutch multi-center community-based cohort of 985 patients demonstrates low risk of neoplastic progression. United European Gastroenterol J 2021;9:929–937.
10. Roumans CAM, Zellenrath PA, Steyerberg EW, et al. Sex differences in neoplastic progression in Barrett's esophagus: a multicenter prospective cohort study. Cancers (Basel) 2022; 14:3240.
11. Chen X, Liu BL, Harpaz N, et al. Aberrant p53 expression is associated with neoplastic progression in Barrett oesophagus diagnosed as indefinite for dysplasia. Histopathology 2023; 82:454–465.

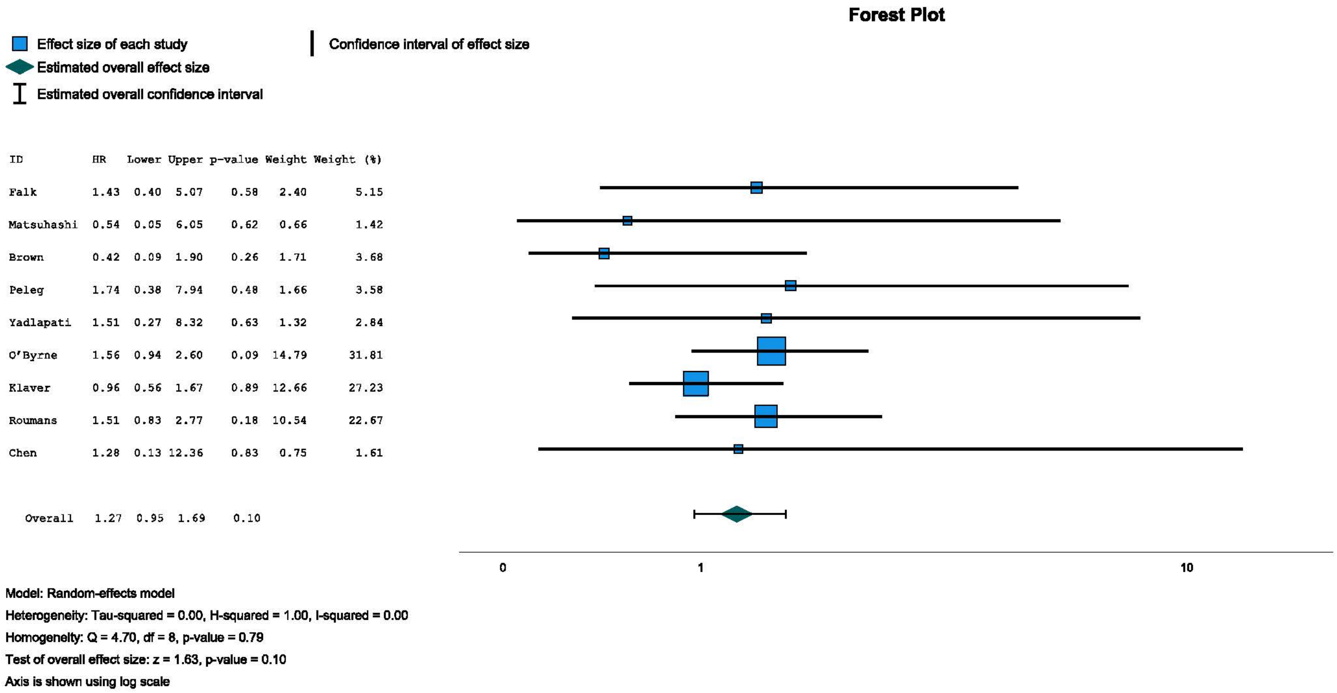

Supplementary Figure 1. Leave-one-out analysis without data from Kambhampati et al[e6]; pooled hazard ratio for neoplastic progression in females versus males with Barrett's esophagus.

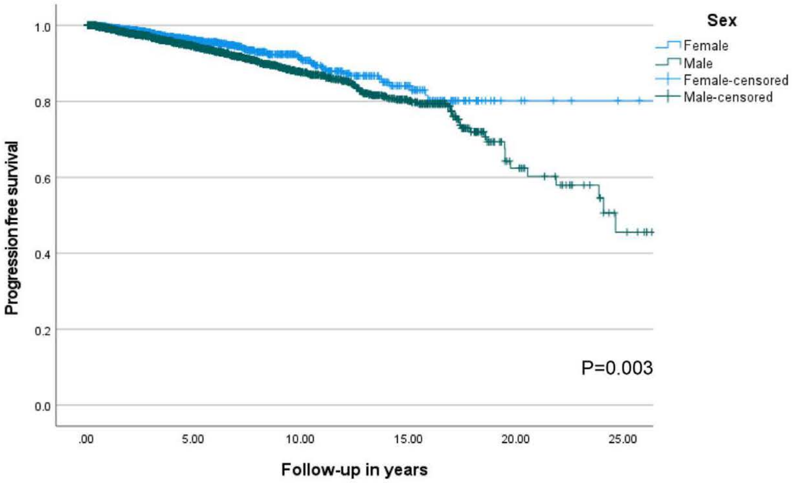

Numbers at risk (number of events)

Females	2196 (55)	769 (21)	224 (12)	78 (3)	8 (0)	3 (1)
Males	4979 (185)	1999 (87)	636 (37)	227 (19)	34 (5)	9 (0)

Supplementary Figure 2. Kaplan-Meier curves for progression-free survival in females vs males.

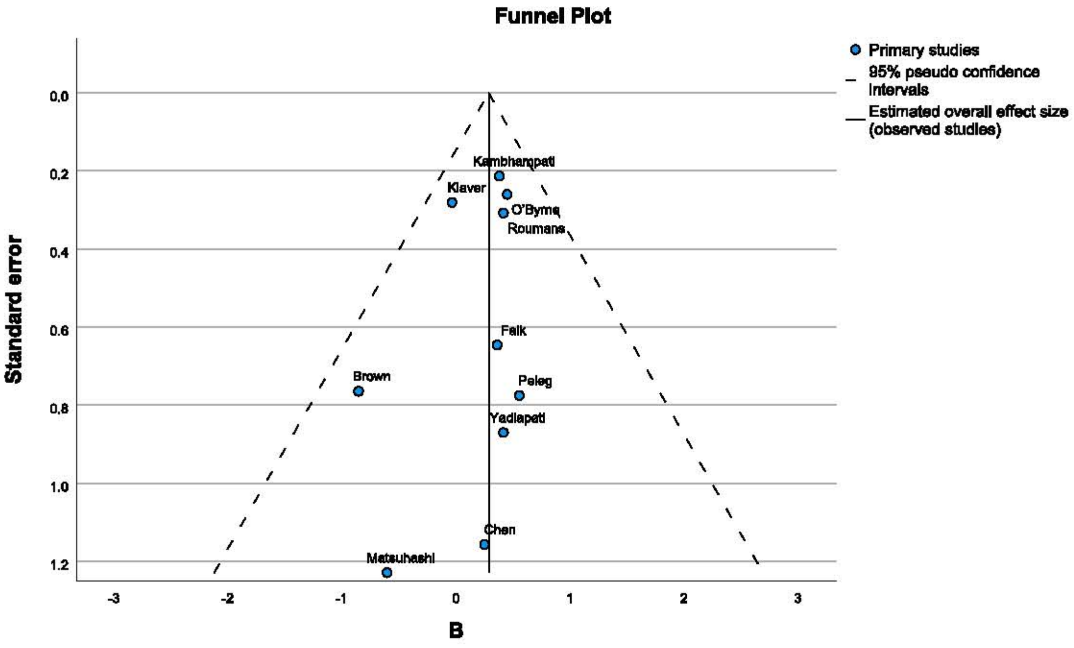

Supplementary Figure 3. Funnel plot.

Supplementary Table 1. Newcastle-Ottawa Scale to Assess the Quality of the Included Studies

	Selection				Comparability	Outcome			
Study	Representative-ness of Exposed Cohort	Selection of Non-exposed Cohort	Ascertainment of Exposure	Outcome not Present at Start of Study	Comparability of Cohorts Based on Design or Analysis	Assessment of Outcome	Length of Follow-Up	Adequacy of Follow-Up	Total
Falk et al, 2005[e1]	*	*	*	*	**	*	*	*	9/9
Matsuhashi et al, 2017[e2]	*	*	*	*	**	*	0	*	8/9
Brown et al, 2018[e3]	*	*	*	*	*	*	*	*	9/9
Choi et al, 2018[e4]	0	*	*	*	*	*	0	0	5/9
Peleg et al, 2021[e5]	*	*	*	*	**	*	0	*	8/9
Kambhampati et al, 2020[e6]	0	*	*	*	**	*	*	*	8/9
Yadlapati et al, 2020[e7]	*	*	*	*	*	*	0	0	6/9
O'Byrne et al, 2020[e8]	*	*	*	*	**	*	0	*	8/9
Klaver et al, 2021[e9]	*	*	*	*	**	*	*	*	9/9
Roumans et al, 2022[e10]	*	*	*	*	**	*	*	*	9/9
Chen et al, 2023[e11]	0	*	*	*	*	*	0	*	6/9

Values are n/n. A study can be awarded a maximum of one star (*) for each numbered item within the Selection and Outcome categories. A maximum of two stars (**) can be given for Comparability. In case no stars are provided, this is reported as a 0. Overall, studies can be awarded with 0–9 stars, with a rating of 0–2 labelled as poor quality, 3–5 as fair quality, and 6–9 as good/high quality studies.

Supplementary Table 2. Egger's Regression-Based Test[a]

					95% Confidence Interval	
Parameter	Coefficient	SE	*t*	Significance (2-Tailed)	Lower	Upper
(Intercept)	0.437	0.2311	1.892	0.095	–0.096	0.970
SE[b]	–0.473	0.6051	–0.782	0.457	–1.868	0.922

[a]Random-effects meta-regression.
[b]Standard error of effect size.
